# Once a man tests, the partner tests as well. A comparison by gender for HCT and STD clinic attendance

**DOI:** 10.1186/1742-4690-9-S1-P113

**Published:** 2012-05-25

**Authors:** Maria Nambira, Lydia Mwolobi

**Affiliations:** 1 Monitoring and Evaluation Specialist at African Medical and Research Foundation, Kampala, Uganda

## Introduction

Since its outbreak in 1986, Uganda has registered a tremendous decline in HIV prevalence rates from 15% in 1991 to 5% in 2002 though up again to 6.4% in 2006 (2006 UDHS). Currently, an estimated 100,000 new HIV infections occur annually. In recent years, the uptake and practice of preventive behavior have declined, particularly among men. Currently, almost 40 %t of people with HIV are not diagnosed until they already have developed AIDS. That can be up to 10 years after they first became infected with HIV. Finding out whether a person is infected with HIV is the first step to improving their health and that of their partners and their families. The study aimed to assess the contribution, role of men in the fight of HIV/AIDS through HCT.

## Methods

The project monitored all individuals attending STD and ART clinics. The project sought to check whether when requested, males brought their female counterparts for testing and vice versa during the September 2010 to Sept 2011 at Luwero health centre IV in Luwero district, Uganda. Clinical data was used.

## Results

In the reporting period, we estimated that 1,230 people were attending ART clinics and about 3,200 people attending STD clinics. More males once tested brought more spouses for testing. The numbers were much less vice versa. For every 1 man tested at least 3 women were tested. This was also recorded in polygamous relationships. Clinic Type Males Females Total Attendants Attendants Who Brought Spouses Males Females STD 1,091 2,109 1,230 567 (52%) 111 (5.3%) ART/HCT 383 847 3,200 146 (38%) 57 (6.7%).

## Conclusion

HCT needs to emphasized in the struggle to reduce HIV incidence and males contribute to this trend setting. Due to the poor health seeking behavior of males compared to females, low HCT is done. The impact of HCT can be greater when men are targeted to attend ART and STD clinics since they have a bigger ability to bring their spouses to test as well. Health facilities should design ways to attract more males for HCT and STDs.

